# Computational Design of Potent and Selective d-Peptide Agonists of the Glucagon-like Peptide-2 Receptor

**DOI:** 10.1021/acs.jmedchem.3c00464

**Published:** 2023-07-25

**Authors:** Pedro A. Valiente, Satra Nim, Jisun Kim, Philip M. Kim

**Affiliations:** † Donnelly Centre for Cellular and Biomolecular Research, University of Toronto, Toronto, Ontario M5S 3E1, Canada; ‡ Department of Molecular Genetics, University of Toronto, Toronto, Ontario M5S 3E1, Canada; § Department of Computer Science, University of Toronto, Toronto, Ontario M5S 3E1, Canada

## Abstract

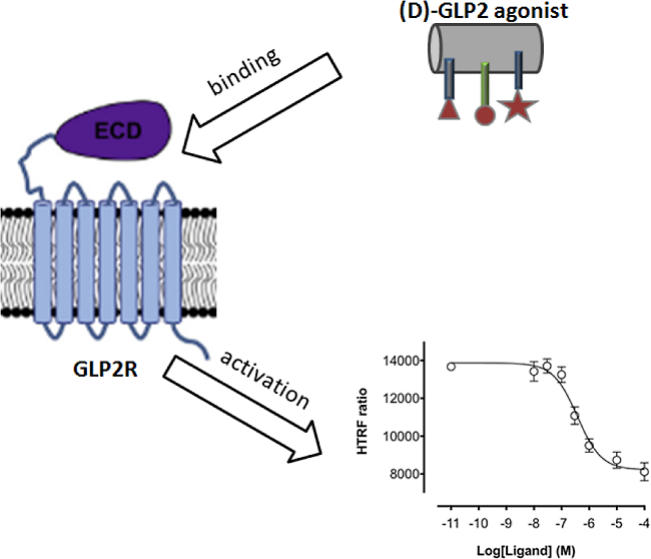

Here, we designed three d-GLP-2 agonists that activated the glucagon-like peptide-2 receptor (GLP-2R) cyclic adenosine monophosphate (cAMP) accumulation without stimulating the glucagon-like peptide-1 receptor (GLP-1R). All the d-GLP-2 agonists increased the protein kinase B phosphorylated (p-AKT) expression levels in a time- and concentration-dependent manner in vitro. The most effective d-GLP-2 analogue boosted the AKT phosphorylation 2.28 times more effectively compared to the native l-GLP-2. The enhancement in the p-AKT levels induced by the d-GLP-2 analogues could be explained by GLP-2R’s more prolonged activation, given that the d-GLP-2 analogues induce a lower β-arrestin recruitment. The higher stability to protease degradation of our d-GLP-2 agonists helps us envision their potential applications in enhancing intestinal absorption and treating inflammatory bowel illness while lowering the high dosage required by the current treatments.

## Introduction

The glucagon-like peptide-2 (GLP-2) belongs to the glucagon-like peptide family, which includes glucagon-like peptide-1 (GLP-1) and glucagon. The pro-glucagon gene encodes GLP-2, which becomes active following post-translational processing by prohormone convertases. The GLP-2 receptor (GLP-2R) is a class B G-protein-coupled receptor (GPCR) subfamily member predominantly expressed in the gut, pancreas, and brain.^1^ The electron microscopic structure of the GLP-2R in a complex with GLP-2 and a Gs heterotrimer was reported recently, and it resembled previous class B GPCRs like the calcitonin gene-related peptide receptor (CGRP-R),^2^ the GLP-1 receptor (GLP-1R),^3^ the corticotropin-releasing factor-1 and -2 receptors (CFR-1R and CFR-2R),^4^ the human glucagon receptor (GCCR),^5^ and the parathyroid hormone receptor-1 (PTH-1R).^6^ However, it showed that GLP-2R employs a novel peptide-recognition mechanism unique to GLP-2 but not to GLP-1 or glucagon.^7^

GLP-2 increases mesenteric blood flow shortly after its release^8,9^ and has been found to modulate gastric secretion in humans.^9,10^ Furthermore, preclinical studies have revealed the role of GLP-2 in intestinal barrier function, enterocyte differentiation, nutrient transport regulation, and inhibition of inflammatory processes.^11−13^ However, like GLP-1, GLP-2 is rapidly degraded in vivo by dipeptidyl peptidase 4 (DPP-IV),^14^ and its short half-life demands regular dosage, limiting its therapeutic applications.^15^

GLP-2-related peptides resistant to DPP-IV inactivation have been developed to treat Crohn’s disease, short bowel syndrome, and other inflammatory disorders.^15^ One of which, known as teduglutide,^16,17^ is available in the market, and others like apraglutide^18^ and glepaglutide^19^ are in the late-stage of clinical development. However, despite having a longer in vivo half-life than natural GLP-2,^15^ all these GLP-2-related peptides still require daily or twice-a-week dosing.^16,19^

Peptides made from d-amino acids are highly resistant to proteolytic degradation, as native proteases can only recognize and cleave proteins comprised of l-amino acids. The retro-inverse (RI) analogues have peptide sequences in a reverse direction to that of natural peptides and chirality of the amino acids inverted from l to d.^20^ These peptides are much less immunogenic than the parent peptides, given their protease-resistance hindering the processing by peptidases and thus their presentation in the MHC complexes in correct lengths.^21^ RI peptides have been used with some success to mimic the topology of unstructured peptides but fail if the peptides have a secondary structure.^20^ We previously created an in-house approach for converting (l)-peptides to highly stable α-helical d-peptides after scanning a mirror-image version of the protein data bank (d-PDB).^22^ Using this methodology, we have developed d-peptide analogues capable of stimulating the GLP-1 and PTH receptors^22^ and blocking the SARS-CoV-2 infection.^23,24^

This manuscript presents the design of selective and potent d-GLP-2 agonists of the GLP-2R by matching the conformations of the crucial hotspots in GLP-2. The most potent variant stimulated HEK293 cells transfected with the GLP-2 receptor with an EC_50_ of 226 nM; this value is 5.7 times less potent than that measured for GLP-2 (EC_50_ = 40 nM). All d-peptides are highly resistant to protease degradation and selectively stimulate the GLP-2 receptor without triggering GLP-1 receptor signaling. Notably, the most effective design boosted the AKT phosphorylation 2.28 times more compared to l-GLP-2. These selective d-GLP-2 agonists are potential lead candidates for improving intestinal absorption and treating inflammatory bowel disease.

## Results

### Computational Design of d-Peptide Agonists of the GLP-2 Receptor

**Figure 1:**
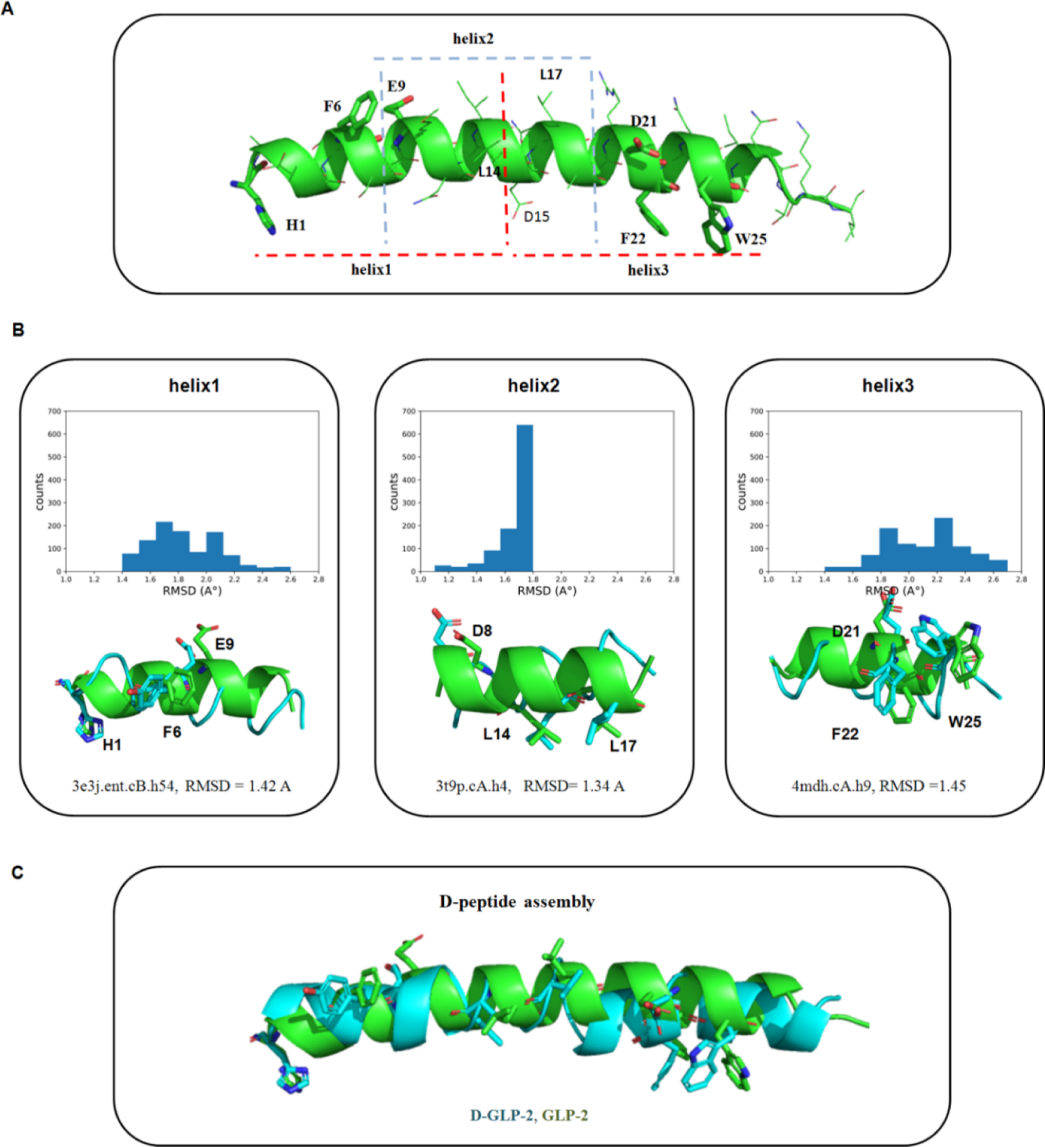
Design strategy of a novel d-peptide agonist of the glucagon like-2 receptor. (A) 3D structure of GLP-2 extracted from the complex with the glucagon like-2 receptor. The GLP-2 structure was divided into three fragments for running the query process: helix1 (H1-L14), helix2 (S7-A19), and helix3 (D15-I31). The dotted lines indicated the length of each helix. For clarity, only the hotspots selected in GLP-2 (H1, F6, D8, E9, L14, L17, D21, F22, and W25) were highlighted as licorice. (B) root mean square deviation (RMSD) profiles of the best output structures of each helix in the d-PDB database search. The structural superposition of the best d-retro-inverted match obtained for each helix was also shown. (C) Assembly of the d-retro-inverted GLP-2 analogue (d-GLP-2, cyan) by joining the three d-peptide matches obtained from the d-PDB database search. Structural superposition of d-GLP-2 over GLP-2 (light green). The hotspots and matching residues in GLP-2 and d-GLP-2, respectively, are highlighted as licorice.

GLP-2 agonists show promising potential in the treatment of patients with intestinal failure associated with short bowel syndrome (SBS-IF). GLP-2 agonism improves intestinal nutrition absorption, while continued therapy is likely required for long-term benefit.^15^ Here, we create new d-peptide agonists of the GLP-2R by matching the conformation of the essential hotspots for GLP-2 activity by scanning a d-PDB database using our in-house method for converting l-peptides to highly stable d-peptide analogues. The GLP-2 helical structure extracted from the complex with the GLP-2R served as the starting point for developing the novel d-GLP-2 agonists. Before searching the d-PDB database, we divided the GLP-2 helix structure into three overlapping fragments denoted as helix1, helix2, and helix3. Helix1 extends from H1 to L14, helix2 from S7 to A19, and helix3 from D15 to I31. We defined H1, F6, and E9 as hotspots in helix1. These residues are located in the GLP-2 region interacting with the GLP-2 receptor’s transmembrane (TM) core. H1 and F6 are conserved, while E9 keeps the negative charge of the equivalent position in GLP-1 or glucagon. For helix2, we designated D8, L14, and L17. D8 is not conserved in GLP-1 and glucagon and primarily interacts with the TM2 and TM7 and the extracellular loop 2 (ECL2). L14 is conserved in GLP-1 and glucagon and binds to TM1 and ECL1, while L17 is not conserved in GLP-1 and glucagon and targets the ECL1. D21, F22, and W25 were chosen in helix3 (Figure 1A). These residues bind to the ECL1 of the GLP-2 receptor. F22 and W25 are conserved in GLP-1 and glucagon, respectively, whereas D21 is conserved in glucagon while retaining the negative charge of the GLP-1 equivalent site. An early experimental study supported our hotspot selection, given that replacing all these residues with alanine reduced the binding and activation of the GLP-2 receptor.^7,25^

By combining different sets of specific atom levels retrieved from the hotspot residues, 36 query structures were created for helix1, 27 for helix2, and 27 for helix3 (Table S1). Several matches were discovered in the d-PDB database after running the search procedure for every helix independently. Figure 1B depicts the RMSD profiles of the 1000 best output structures from the search for each helix. The RMSD between the specific atoms in each query structure and the matched structures at d-PDB was used to evaluate the match quality (Figure 1B). The best match for helix1 was found in 3E3J at 1.42 A, for helix2 in 3T9P at 1.34 A, and for helix3 in 4MDH at 1.45 A (Figure 1B). We next combined the best matches to create the d-GLP-2 peptide analogue. Notably, after the d-peptide-assembling step, the high pairing between the hotspots and matching residues in GLP-2 and d-GLP-2 was maintained (Figure 1C).

**Figure 2:**
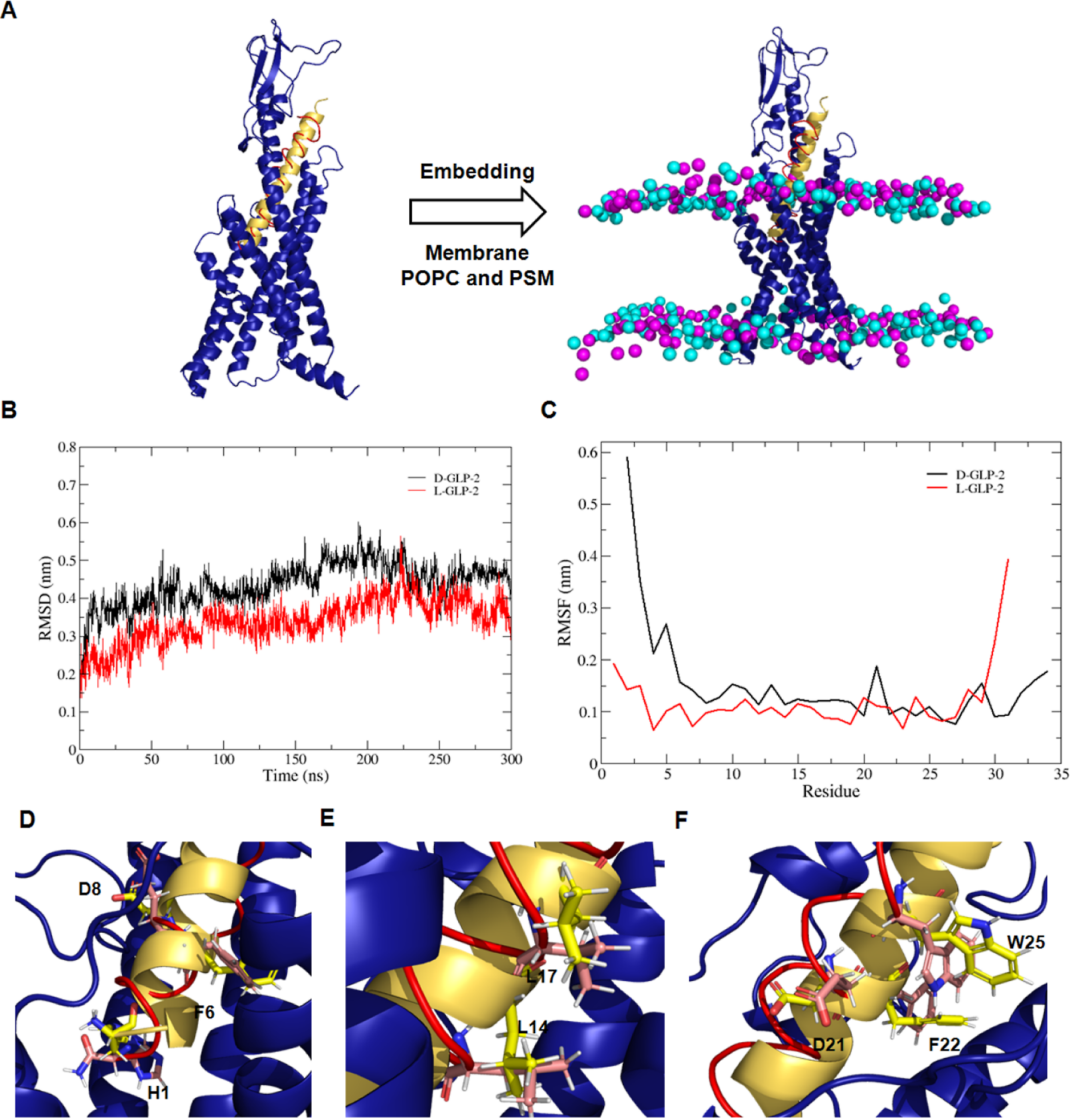
Modeling the 3D structure of the GLP-2 receptor bound to d-GLP-2. (A) Structural superposition of d-GLP-2 (red cartoon) over the 3D structure of GLP-2 (yellow cartoon) bound to the GLP-2R (blue cartoon). The 3D structure of the GLP-2R in complex with the peptides was embedded in a POPC/PSM membrane with a composition of 1:1. The P atoms in the phospholipid polar heads are colored in cyan (POPC) and magenta (PSM). (B) RMSD of the heavy atoms of d-GLP-2 and GLP-2 bound to the GLP-2R along the MD simulations. (C) RMSF per residue of the heavy atoms of d-GLP-2 and GLP-2 bound to the GLP-2R along the MD simulations. The most representative cluster extracted from the MD simulations of the d-GLP-2 (red) and GLP-2 (yellow) bound to the GLP-2R (blue) were superimposed. (D) Zoomed image of the structural alignment at helix1. (E) Zoomed image of the structural alignment at helix2. (F) Zoomed image of the structural alignment at helix3. The hotspots (yellow) and matching (red) residues were highlighted as licorice. The nitrogen and oxygen atoms were colored blue and red, respectively, in the hotspots and matching residues.

Next, we modeled the 3D structure of the GLP-2R in the complex with d-GLP-2 by superimposing the d-peptide analogue onto the GLP-2R structure bound to GLP-2 (PDB code: 7D68). The extracellular domain of the GLP-2R was modeled using the GLP-1 receptor as a template (5VAI). We then embedded the resulting model in a 1-palmitoyl-2-oleoylphosphatidylcholine (POPC)/palmitoylsphingomyelin (PSM) (1:1) bilayer before evaluating its binding mode stability using 300 ns MD simulations (Figure 2A). As a control, we simulated GLP-2R bound to wild-type l-GLP-2. The calculated RMSD profiles for both peptide’s heavy atoms revealed that d-GLP-2 quickly stabilized in a new equilibrium position close to the initial structure, like l-GLP-2 (Figure 2B). The calculated root mean square fluctuation (RMSF) profiles revealed that the C-terminal segment of d-GLP-2 shares a similar stability compared to the N-terminal region of l-GLP-2 (Figure 2C).

d-GLP-2 has a reversed orientation when compared to l-GLP-2. This difference in direction means that d-GLP-2 has its C-terminal residue embedded in the GLP-2R’s TM domain, whereas l-GLP-2 has its N-terminal residue interacting with the receptor’s extracellular domain. The structural superposition of the most representative clusters extracted from the MD simulation of the GLPR2 + d-GLP-2 and GLPR2 + l-GLP-2 complexes revealed a significant matching between the specific residues in d-GLP-2 and the majority of the hotspots defined in l-GLP-2 (Figure 2D–F). The most significant difference was observed for H1, the N-terminal residue of l-GLP-2 (Figure 2D).

**Figure 3:**
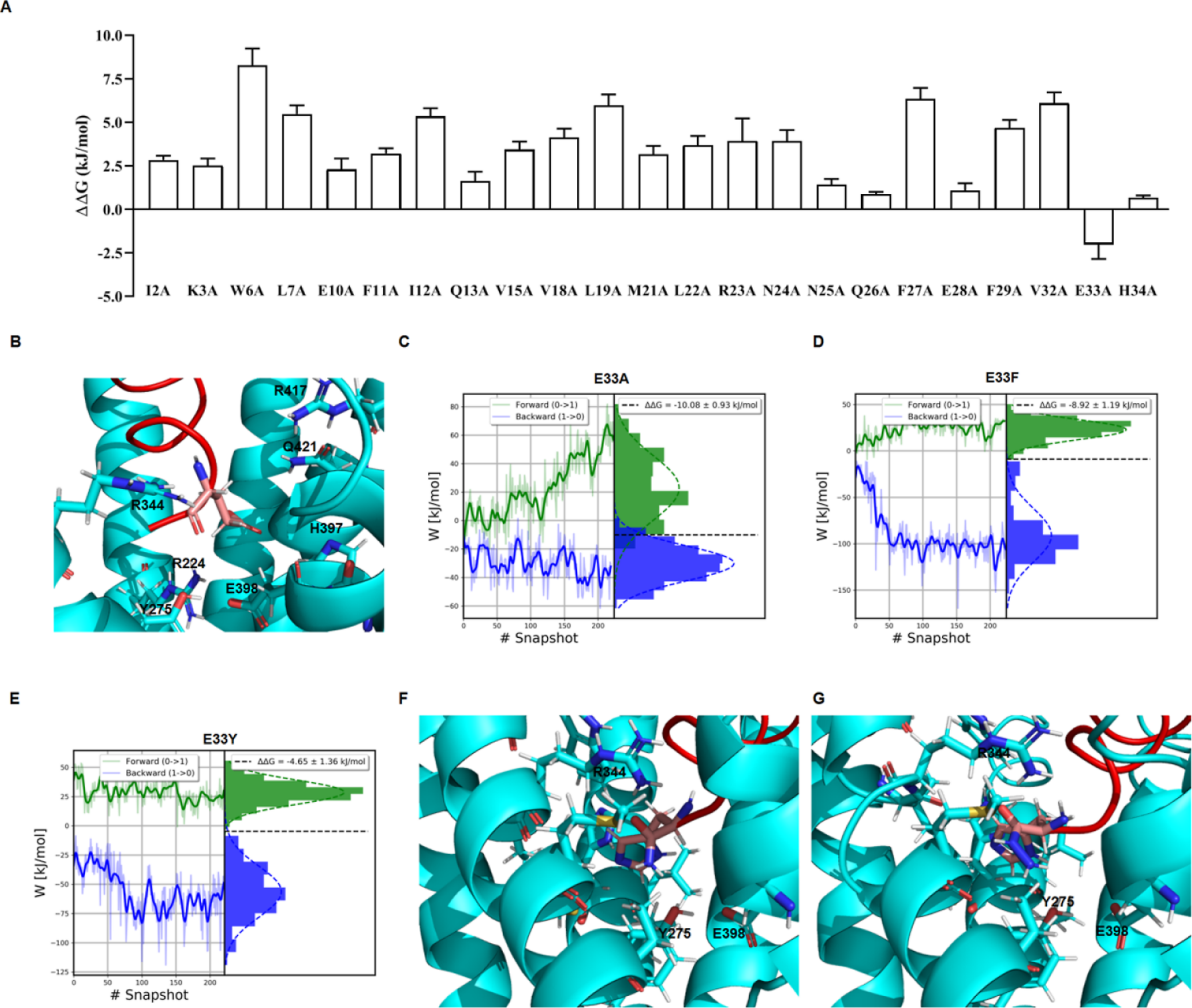
Computational redesign of d-GLP-2. (A) Computational alanine scanning over the MD simulation of the GLP-2R + d-GLP-2 complex using the MM/GBSA method. The binding free energy differences were calculated as ΔΔ*G* = Δ*G*_mutant_ – Δ*G* wild type, where ΔΔ*G* > 0 indicates a favorable contribution to binding affinity. In contrast, ΔΔ*G* < 0 shows an unfavorable contribution to the complex formation. (B) Zoomed image of the binding interaction of position E33 in d-GLP-2 with the GLP-2R. (C) Predicting the change in the binding affinity of d-GLP-2 of mutation E33A. (D) Predicting the change in the binding affinity of d-GLP-2 of mutation E33F. (E) Predicting the change in the binding affinity of d-GLP-2 of mutation E33Y. We used the CGI method combined with the dual-system single-box approach to calculate the binding free energy of the three mutations. (F) Zoomed image of the binding interaction of position H34 in d-GLP-2 modified with the NH2 group with the GLP-2R. (G) Zoomed image of the binding interaction of position H34 in d-GLP-2 modified with the NH–NH2 group with the GLP-2R.

Following that, we performed a computational alanine scanning with the molecular mechanics/generalized Born surface area (MM/GBSA) method to investigate the contribution to the binding affinity for the GLP-2R of different positions within d-GLP-2. As anticipated, most of the residues in the d-peptide analogue structurally aligned with the hotspot residues in GLP-2 that significantly contribute to the binding affinity with the GLP-2R. However, an important exception was H34, the C-terminal residue matching with the N-terminal His of GLP-2. On the other hand, E33 was the only predicted position that negatively impacted d-GLP-2’s binding affinity (Figure 3A). The proximity of E33 to E398 in the GLP-2R TM domain could explain this residue’s negative contribution (Figure 3B). We then investigated the impact of replacing E33 with hydrophobic or aromatic residues like Ala, Phe, or Tyr to destabilize this negative interaction. We used the Crooks Gaussian intersection (CGI) approach to estimate how much the point mutation E33A, E33F, and E33Y will improve the binding strength of d-GLP-2 for the GLP-2R. The free energy calculations indicated that all the mutations will increase the design’s binding affinity (Figure 3C–E). We chose the mutation E33A for further experimental validation because of its improved affinity and calculation convergence. Given the d-peptides’ reversed orientation compared to that of l-GLP-2, we hypothesized that adding a hydrazide moiety (NH–NH2) at the C-terminal residue could boost their activity (Figure 3F,G).

### Design d-GLP-2 Agonists Activate Specifically the GLP-2R

**Figure 4:**
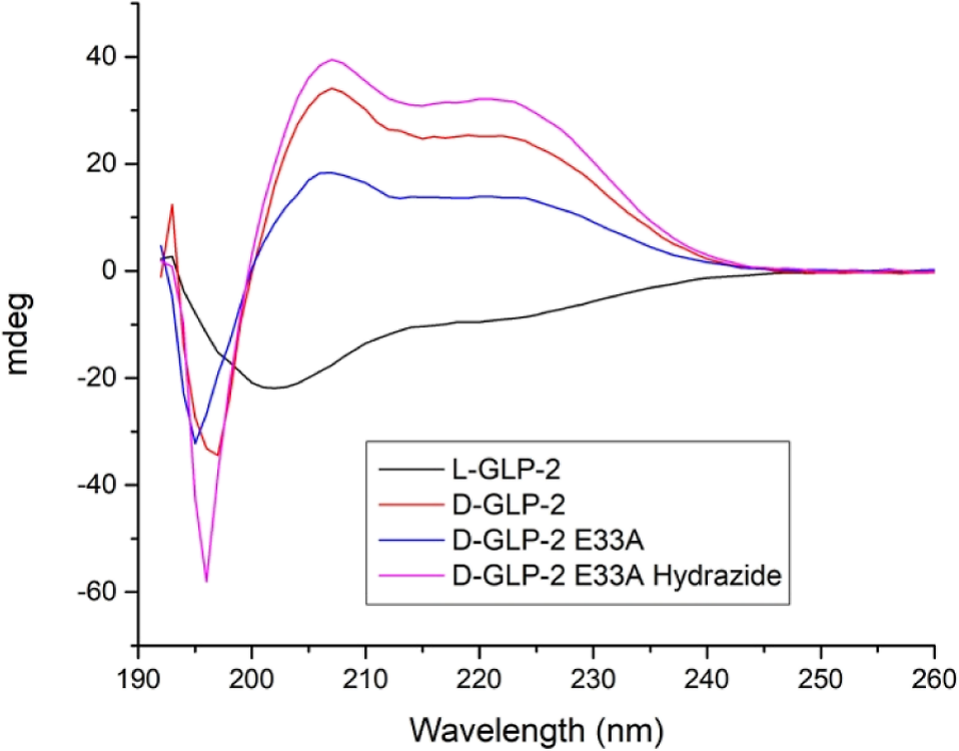
CD measurements of the designed d-peptides and l-GLP-2 in solution. Peptide concentrations were 20 μM for l-GLP-2 and 150 μM d-GLP-2 in acetonitrile/PBS (1:2). Secondary structure determination was carried out using a Jasco J-720 spectro-polarimeter. Samples were read using a 0.1 cm cuvette path length with three accumulations per run and 50 nm/min scanning speed.

First, we performed circular dichroism (CD) measurements of the peptides in solution to determine the peptides’ secondary structure. According to our findings, all d-peptides remain helical in solution. Except for d-GLP-2 E33A, most of the d-peptides showed a higher helical content than l-GLP-2 (Figure 4).

**Figure 5:**
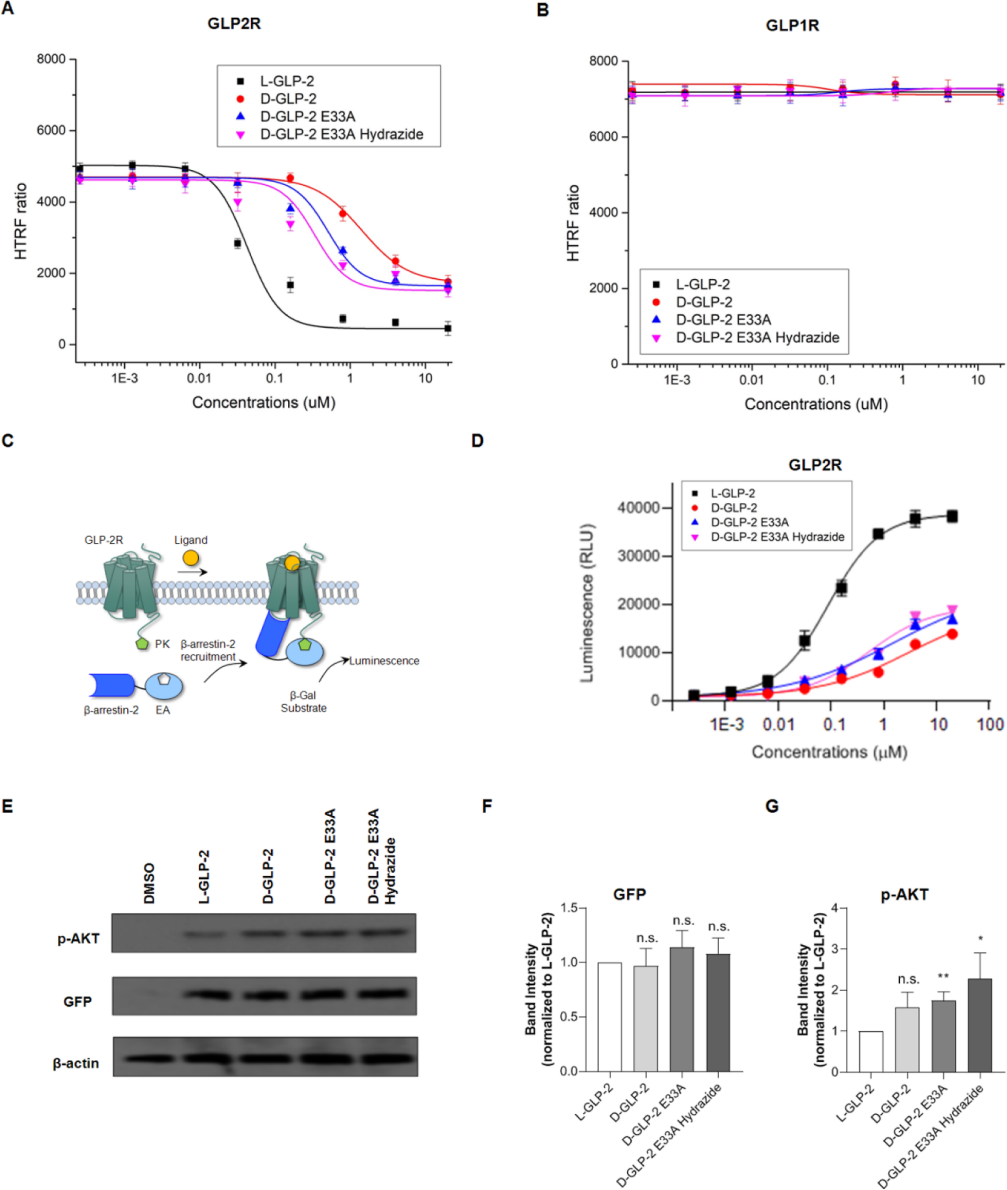
Experimental validation of the d-GLP-2 analogues. (A) Activity profile of l-GLP-2 and the design d-peptides over HEK293 cells stably expressing GLP-2R and CRE-luciferase. (B) Activity profile of l-GLP-2 and the design d-peptides over control HEK293 cells stably expressing GLP-1R and CRE-luciferase. (C) A schematic illustration of the β-arrestin-2 recruitment assay. The reporter cells co-expressed the GLP-2R tagged with ProLink (PK) and β-arrestin-2 tagged with enzyme acceptor (EA). Upon activation of the GLP-2R-PK, there is recruitment of β-arrestin-2-EA, which in turn led to the complementation of the two β-galactosidase enzyme fragments (EA and PK), thereby hydrolyzing the substrate to generate a chemiluminescent signal. (D) Dose-response curve for β-arrestin-2 recruitment of GLP-2R by l-GLP-2 and the design d-peptides. (E) Western blots showing the GFP and protein kinase B phosphorylated (p-AKT) expression levels induced by the d-GLP-2 analogues and the native l-GLP-2 at 10 μM. We measured the GFP expression at 36 h, while the p-AKT expression was evaluated at 3 h. We measured the β-actin expression in all the treatments as loading controls. (F) Quantification of the GFP expression for all the peptides relative to the l-GLP-2-treated cells after 36 h of incubation. (G). Quantification of the AKT phosphorylation induced for all the peptides relative to the l-GLP-2-treated cells after 3 h of incubation. In (FandG) *P < 0.05, **P < 0.01 versus l-GLP-2-treated cells; n.s., not significant.

We next created a stable GLP-2R-expressing HEK293 cell line to determine the capacity of l-GLP-2 and the d-peptide designs to activate the GLP-2R. GLP-2 binding to the GLP-2R has previously been reported to activate adenylyl cyclase, resulting in the generation of cAMP, which stimulates protein kinase A (PKA) that plays an essential role in a variety of downstream cellular processes. To assess the potency of l-GLP-2 and the d-peptides, we used the homogeneous time-resolved fluorescence (HTRF) cAMP assay. This methodology identifies intracellular cAMP by competing for the anti-cAMP antibody with d2-labeled cAMP following cell lysis. The Förster resonance energy transfer (FRET) signal is then disrupted as intracellular cAMP levels rise. l-GLP-2 decreased the FRET signal in GLP-2R expressing HEK293 cells with a half maximal effective concentration (EC_50_) value of 40.4 nM. d-GLP-2 reduced the FRET signal to a lesser extent, with an EC_50_ of 1417 nM. The mutation E33A improved the activation of the GLP-2R by d-GLP-2 E33A by 3.4 fold (EC_50_ = 414 nM) compared to d-GLP-2, while adding the hydrazide moiety at the C-terminal of this mutant peptide enhanced the activity of d-GLP-2 E33A hydrazide by 6.26 fold (EC_50_ = 226 nM). All d-GLP-2 agonists stimulate the GLP-2R with efficacies ranging from 64.5 to 69.4% compared to l-GLP-2 activation (Figure 5A).

We next tested the specificity of the d-GLP-2 analogues to activate the GLP-1R using a stable GLP-1R-expressing HEK293 cell line. Notably, none of the d-GLP-2 analogues, nor the l-GLP-2 peptide could activate the GLP-1R signaling, demonstrating their unique GLP-2R recognition (Figure 5B). We chose the GLP-1R for the specificity assays among the different members of the B-class GPCR receptor subfamily because of its higher percentage of sequence identity with the GLP-2R.^26^ Given their inability to stimulate the GLP-1R, we reasoned that these analogues would not activate other members of this subfamily.

Following that, we measured the β-arrestin 2 recruitment induced by the d-GLP-2 analogues and l-GLP-2 via a previously reported β-galactosidase complementation method (Figure 5C).^27^l-GLP-2 recruited β-arrestins with an EC_50_ value of 88.7 nM, while the d-GLP-2 analogues’ potencies and efficacies ranged from 524.6 to 3031 nM and 34.6 to 47.8%, respectively. Remarkably, the recruitment of β-arrestins for d-GLP2_E33A_hydrazide (EC_50_ = 524.6 nM) is reduced by 5.9 fold compared to l-GLP-2 (Figure 5D).

We then investigated the downstream effects of stimulating GLP-2R with the d-GLP-2 analogues to study the d-peptide designs’ effects on GLP-2R. We wanted to determine whether activating GLP-2R with d-GLP-2 analogues would promote GFP expression in HEK293 cells containing a GFP gene under the control of cAMP response element binding protein (CREB) and AKT phosphorylation. We also checked the beta-actin expression in all the treatments as loading controls. Of relevance, all peptides induced a similar GFP expression under the CREB, reaching saturation between 24 and 48 h of incubation (Figures 5E,F, S1A, Table S3). We also found a concentration-dependent increase in GFP expression in response to all the peptides’ stimulation (Figure S1B).

Remarkably, all d-GLP-2 analogues triggered higher phosphorylation levels of AKT than l-GLP-2 in HEK293 cells expressing GLP-2R (Figure 5E,G). We quantified the relative area of the p-AKT band detected after stimulating the HEK293 cells stably expressing the GLP-2R with the peptides. We used l-GLP-2 treatment as the reference. We found that the d-GLP-2 analogues increased p-AKT expression levels in a time-dependent manner, reaching the highest level at 3 h (Figure S1C, Table S2). We also detected a concentration-dependent increase in p-AKT levels in response to all the peptides’ stimulation (Figure S1D). d-GLP-2 increased the level of p-AKT by 1.58 ± 0.37 times, while introducing the mutation E33A increased the p-AKT level by 1.74 ± 0.21 times. Notably, adding the hydrazide moiety at the C-terminal of d-GLP-2 E33A enhanced the p-AKT level by 2.28 ± 0.68 times (Figure 5G, Table S2). It is well-known that AKT is recruited and activated by the PI3K through the action of phosphatidylinositol 3,4,5-trisphosphate (PIP3), a second messenger that amplifies the signal.

### High Resistance of d-GLP-2 Agonists to DPP-IV and Proteinase K Degradation

**Figure 6:**
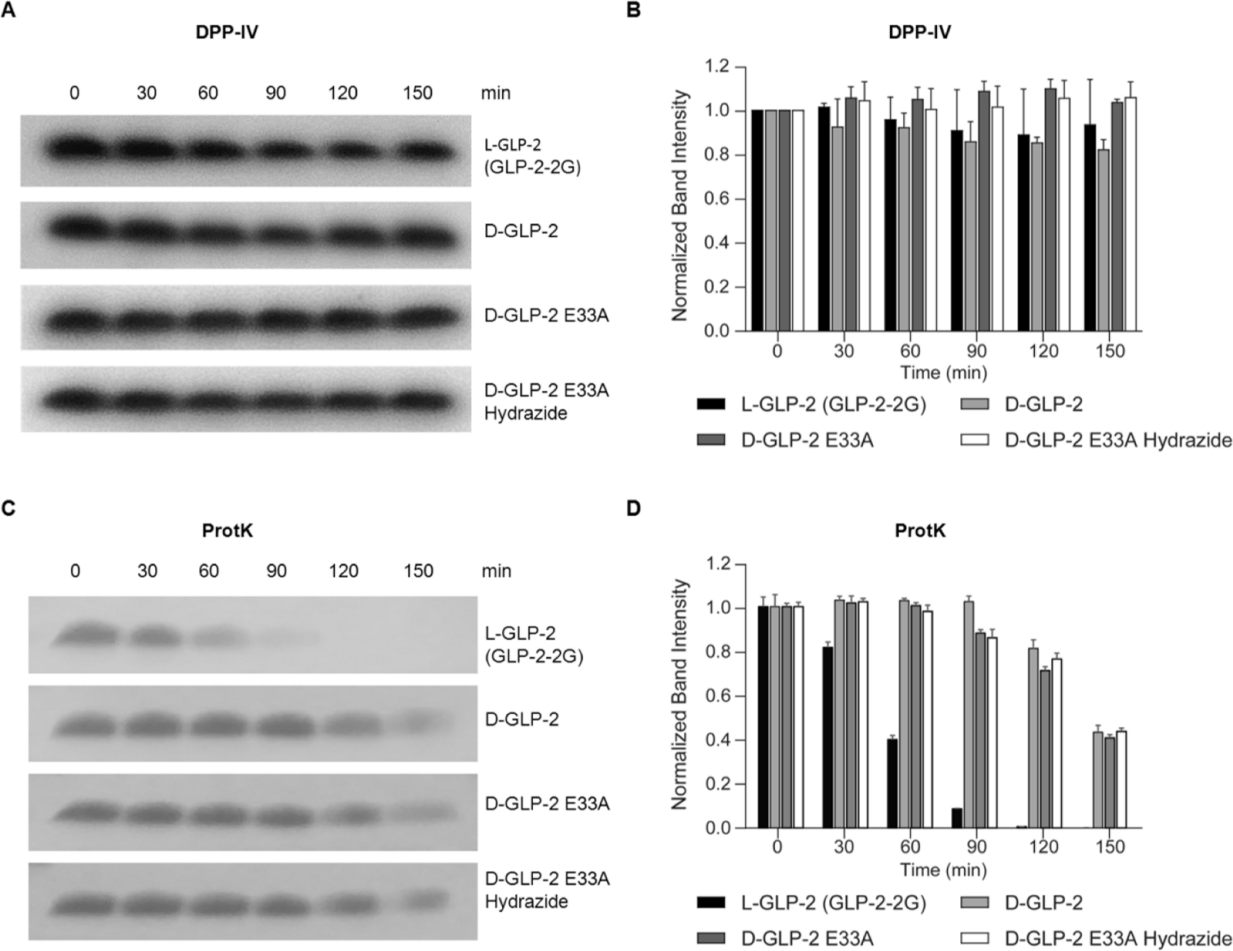
The design d-GLP-2 analogues are resistant to DPP-IV and proteinase K degradation. (A) Sample gel images of the designed d-peptides and l-GLP-2 treated with DPP-IV over 150 min. Gels were stained with Coomassie brilliant blue dye. Band densitometries were calculated using ImageJ with background subtraction. (B) Quantification of the remaining peptides after DPP-IV treatment at 30 min intervals. Intensities of peptide bands were normalized to the intensity of the untreated peptide (T0). (C) Sample gel images of the designed d-peptides and l-GLP-2 treated with proteinase K over 150 min. Gels were stained with Coomassie brilliant blue dye. Bands densitometries were calculated using ImageJ with background subtraction. (D) Quantification of the remaining peptides after proteinase K treatment at 30 min intervals. Intensities of peptide bands were normalized to the intensity of the untreated peptide (T0).

We next examined the resistance of the d-GLP-2 analogues to DPP-IV degradation. An early report showed that GLP-2_1–33_ was degraded to a shorter form (GLP-2_3–33_) by DPP-IV following incubation with human placental DPP-IV or rat serum but not by serum from DPP-IV-deficient rats.^14^ We used as control, l-GLP-2-2G, a variant with the mutation A2G that is resistant to DPP-IV inactivation. Resistance to protease degradation makes d-peptides appealing for therapeutic applications since it translates into a longer half-life in serum. As expected, the d-GLP-2 analogues remained intact after being incubated with DPP-IV for 2.5 h, similar to l-GLP-2-2G (Figures 6A,B, S2). We then evaluated the resistance to proteinase K (ProtK) degradation, a protease with a broader cleavage sequence than DPP-IV. We observed the almost complete loss of l-GLP-2-2G in 2 h, while more than 70% of the d-GLP-2 analogues lasted after 2 h, and over 40% could still be detected after 2.5 h of ProtK exposure (Figure 6C,D).

## Discussion

GLP-2 analogues resistant to DPP-IV inactivation are required for treating disorders, where intestinal absorption is inefficient or the gut barrier is down-regulated due to an inflammatory process.^15^ Here, we described the development of three d-GLP-2 agonists which activated HEK293 cells stably expressing GLP-2R. To create the novel d-GLP-2 agonists, we scanned our d-PDB database for similar conformations of previously known critical hotspots for l-GLP-2 binding and activation.^7,25^ Hotspot residues play an essential role in molecular recognition, receptor activation, and drug development.^25^ The d-GLP-2 analogues bound the receptor in a RI orientation and matched the configurations of crucial hotspots in GLP-2 function.^7,25^ Significantly, we confirmed that the GLP-2 sequence could be entirely altered except for the hotspots to retain the function. The parent d-GLP-2 agonist activated GLP-2R signaling with an EC_50_ 35 times weaker than that of the native l-GLP-2. To better understand the d-peptide agonist’s lower potency and efficacy than GLP-2, we compared the 3D structures extracted from the MD simulations of the GLP-2R bound to each peptide. Notably, H1, the N-terminal residue of l-GLP-2, has the greatest structural difference between the hotspots and matching residues (Figure 2D). Histidine, at position 1, is essential for signal transduction in several members of the glucagon peptide superfamily. An early study found that the His1Ala substitution in GLP-2 did not significantly impact binding affinity in a screening assay but decreased cAMP production.^25^ Similarly, hGLP-2 analogues with amino-terminal extensions showed lower activation of the GLP-2 receptor.^26^

We also predicted that E33 has a detrimental impact on the d-GLP-2’s binding affinity through CAS (Figure 3A). E33’s unfavorable contribution could be supported by the closeness of this residue to the E398 within the receptor TM domain (Figure 3B). We then explored the benefit of the mutation E33A and the addition of an azide group (NH–NH2) at the C-terminus of d-GLP-2 to boost the potency and efficacy of the d-peptide agonists. The most successful design triggered the GLP-2R activity with an EC_50_ of 226 nM, which is 5.7 times less potent than the native GLP-2 (EC_50_ = 40 nM). Intriguingly, it only partially activated the GLP-2R with 69.4% efficacy compared to the l-GLP-2 activation. Remarkably, the effectiveness of all our designs was significantly higher than the 11% efficacy, recently measured for GLP-2_3–33_ by Gadgaard et al.^28^ GLP-2_3-33_, the product of DPP-IV inactivation, acted as a competitive antagonist of GLP-2_1–33_ and could not recruit β-arrestin 1 and 2.^28^

Radioligand binding is widely used to determine the known ligands’ affinity for GPCR receptors.^29^ First, saturation experiments could be used to determine the binding strength of increased concentrations of the labeled variants of the d-peptides designed for HEK293 cells expressing the GLP-2R or GLP-1R. The equilibrium dissociation constant (*K*_d_), receptor density (*B*_max_), and Hill slope (*n*_H_) will be measured by fitting to non-linear equations. Next, the affinity and selectivity of the unlabeled d-peptides could be quantified through competition binding assays using a fixed concentration of the labeled d-peptides to a receptor.^29^ Further binding studies will be required to determine the binding affinity of the d-GLP-2 peptides.

In recent years, the kinetics of drug binding and unbinding have been recognized as crucial to the effectiveness and safety of many drugs.^30^ The ligand efficacy at specific GPCRs correlates better with residence time rather than with binding affinity.^31^ Binding kinetics are affected by receptor dynamics because conformational changes are frequently necessary for ligand binding and unbinding.^30^ Further kinetic studies will be required to determine the residence time and increase the efficacy of the d-GLP-2 agonists.

We anticipated the development of highly specific d-GLP-2 analogues by selecting non-conserved residues (D8 and L17) in GLP-2’s central region as hotspots. An early alanine scanning study showed the critical role of L17 in the GLP-2R activation.^25^ Remarkably, all our d-GLP-2 agonists could not trigger the GLP-1R signaling demonstrating their unique GLP-2R specificity. According to a recent study, GLP-2’s middle segment (7–19) differs from that of GLP-1, mainly interacting with the extracellular loop 1 (ECL1) and the TM helices (TM) 1 and 7. This region is critical for GLP-2R’s ligand specificity.^7^ These authors demonstrated that the GLP-2R-mediated cAMP accumulation was nearly fully reduced when the GLP-2 central region was replaced with the equivalent one in GLP-1.^7^ Furthermore, replacing GLP-2R-ECL1 with poly-alanine abrogated GLP-2’s capacity to activate a cAMP response, while replacing it with the GLP-1R equivalent sequence lowered its potency by 196 times.^7^ We envisioned the design of novel d-peptides with dual GLP-1/GLP-2 agonism for treating short bowel syndrome by matching conserved residues in GLP-1 at the middle segment of GLP-2.^15^ Further experiments will be needed to test this hypothesis in the future.

We next compared the downstream effects of stimulating the GLP-2R with the three d-GLP-2 agonists and l-GLP-2 using short-term and long-term stimulation experiments. As a short-term study, we measured the increase of the p-AKT levels after the GLP-2R stimulation for 6 h. The d-GLP-2 analogues increased the p-AKT levels in a time- and dose-dependent manner (Figure S1). The enhancement in the p-AKT levels induced for the d-GLP-2 analogues could be explained by the GLP-2R’s more prolonged activation, given that the d-GLP-2 analogs induce a lower β-arrestin recruitment (Figure 5D). A recent study showed that biased GLP-2 agonists, with strong G protein-coupling but impaired β-arrestin recruitment and receptor desensitization, enhance intestinal growth in mice.^32^ These authors also found that the GLP-2R could be internalized without β-arrestins but with lower efficiency and speed than with the presence of β-arrestins.^32^ Arrestins are considered to be the central regulators of GPCR endocytosis because they bind to both phosphorylated receptors and adaptor protein 2 (AP-2) or clathrin, attracting receptors to clathrin-coated pits to promote internalization.^33^ As a long-term stimulation study, we detected the GFP expression under the control of CREB during 48 h. The more prolonged activation of the GLP-2R compensates for the lower levels of cAMP induced by the d-GLP-2 analogues, making the d-GLP-2 analogues induce similar GFP expression levels as l-GLP-2.

An early study showed that GLP-2′s anti-inflammatory actions were mediated by enteric neural pathways.^34^ GLP-2 activated enteric neurons and increased the number of cells expressing the vasoactive intestinal peptide (VIP) in the submucosal plexus of the ileum. GLP-2 treatment reduced levels of pro-inflammatory cytokines (IFN-γ, TNF-α, and IL-1β) and inducible nitric oxide synthase, with increased levels of IL-10 in ileitis and colitis models. Significantly, the anti-inflammatory effects of GLP-2 were abolished by the co-administration of GLP-2 with a VIP antagonist.^35^ GLP-2 induced VIP expression in enteric neurons via phosphatidylinositol 3-kinase-γ (PI3K) signaling.^36^ PI3K recruited and activated AKT through the action of phosphatidylinositol 3,4,5-trisphosphate (PIP3), a second messenger that amplifies the signal.

The higher increase in the p-AKT expression levels induced by the d-GLP-2 designs compared with the native GLP-2 could be translated into improved anti-inflammatory effects. AKT directly phosphorylates the TSC1/TSC2 complex to inactivate it, and thus, activate mTORC1.^37^ AKT also signals mTORC1 in a TSC1/TSC2-independent way by phosphorylating it and causing its dissociation from mTORC1 inhibitors.^38^ The activation of mTORC1 inhibits the production of pro-inflammatory cytokines such as IL-12, IL-23, IL-6, and TNF-α by inhibiting the transcription factor NF-κB activity. mTORC1 stimulation triggers the expression of anti-inflammatory cytokines such as IL-10 or TGF-β and type I interferons in macrophages.^37^ These activities are mediated by the signal transducer and activator of transcription 3 (STAT3) and the interferon-regulated factors (IRF)-5 and IRF-7.^37,39^ It is well known that upon GLP-2 binding, the GLP-2R can couple with several G protein subunits and activate multiple biochemical pathways.^40^ More research will be needed to determine the effect of our designs on other GLP-2R signaling pathways and their potential applicability as lead candidates for enhancing intestinal absorption and treating inflammatory bowel illnesses. We envisioned the lipidation of these peptides to improve their delivery to the vicinity of the GLP-2 receptors in vivo. A recent study showed that a GLP-2 highly active variant (palmitoylated at position 20) with low β-arrestin recruitment and a half-life of 9.5 h in rats showed improved gut and bone tropism with the increased weight of the small intestine.^28^

## Conclusions

In this work, we designed three d-GLP-2 agonists that activated the GLP-2R cAMP accumulation without inducing GLP-1R stimulation. The most successful design triggered the GLP-2R activity with 30% less efficacy but with 5.7 times less potency than that measured for the native l-GLP-2. Significantly, this d-GLP-2 analogue boosted the AKT phosphorylation by 2.28 fold compared to l-GLP-2. The enhancement in the p-AKT levels induced for the d-GLP-2 analogues could be explained by the GLP-2R’s more prolonged activation, given that the d-GLP-2 analogues induce lower β-arrestin recruitment. The improved stability to protease degradation of our d-GLP-2 agonists helps us envision their potential application in enhancing intestinal absorption and treating inflammatory bowel illness, while lowering the high dosage required by the current treatments.

## Experimental Section

### Design Strategy of the d-GLP-2 Agonists

The query process for each helix segment was run independently using the most recent update of the d-PDB database that our group had previously generated.^22^ GLP-2 hotspot residues were selected based on previous experimental data using as starting structure the GLP-2R in complex with GLP-2 (7D68). Click was used to perform structural alignments between specific atoms in each query structure and each entry in the d-PDB database.^41^ Click uses the molecule coordinates to align groups of atoms independent of the residue order. The matched (d) hotspots from each retrieved helix were aligned with their corresponding (l)-hotspots. The best-retrieved helices for each segment were assembled into d-peptide analogues over the GLP-2 receptor structure using Chimera.^42^

### Molecular Dynamics Simulations

All the initial structures and topology files for the molecular dynamics (MD) simulations of the GLP-2R in complexes with different peptides embedded into a POPC/PSM (1:1) bilayer were built using the membrane builder generator implemented in the CHARMM-GUI web server.^43^ The GROMACS software package^44^ version 2019.3 was used to perform the MD simulations of the GLP-2R + peptide complexes using the CHARMM36-m force field^45^ and the TIP3P water model.^46^ Two consecutive energy minimization (EM) schemes were used to initially relax the systems. The systems were then equilibrated in two sequential *NVT* [constant number of particles (N), constant volume (V), and constant temperature (T)] ensemble simulations before being equilibrated in five successive *NPT* [constant number of particles (N), constant pressure (P), and constant temperature (T)] ensemble simulations at *p* = 1 bar and *T* = 310 K. We gradually released the position restraints that had been applied to the protein-heavy atoms in both steps. Finally, the production *NPT* runs were performed for 300 ns for each system.

### Free Energy Calculations

The gmx_MMPBSA program was used for all MM/GBSA free energy calculations unless stated otherwise.^47^ In all cases, we followed the single-trajectory approach, in which the trajectories for the receptor and the ligand are extracted from that of the complex. Following the protocol recently described by Valdés-Tresanco et al.^48^

We set up free energy calculations with the CGI protocol using the dual system single-box approximation to predict the effect of different mutations in d-GLP-2 on the d-peptides’ binding ability. Briefly, in the dual-system single-box setup, a wild-type GLP-2R complex embedded into a POPC/PSM (1:1) bilayer is positioned in the same box with a solvated unbound mutant d-peptide (λ = 0). The other end-state (λ = 1) contains a mutant d-GLP-2 bound to a GLP-2R embedded into the POPC/PSM bilayer with a solvated wild-type d-GLP-2. To prevent an interaction between the solvated d-GLP-2 and the GLP-2R + peptide/POPC/PSM complex due to motions during the simulation, position restraints were applied at the backbone atoms of the d-GLP-2Met19. Simulation topologies and input files were generated for CHARMM36-m force field^45^ with the pmx package.^49^ For each state (λ = 0 and λ = 1), equilibrium MD simulations of 100 ns length were conducted using the simulation parameters described previously.^50^ From each trajectory, the first 10 ns were discarded; snapshots were taken every 400 ps, and short nonequilibrium thermodynamic integration runs (500 ps) were performed, in which λ was switched from 0 to 1 or from 1 to 0, respectively. The derivative of the Hamiltonian with respect to λ was recorded at every step and the alchemical free energy for the transition was calculated according to Goette and Grübmueller.^51^

### Peptide Synthesis

All peptides were synthesized, purified, and characterized by Lifetein LLC. All peptides’ purity is higher than 95%. In the Supporting Information material, we have provided the details about the characterization of these peptides [molecular weight, purity, high pressure liquid chromatography (HPLC) and mass spectra (MS)] (Figures S3–S5, Table S3).

### CD Measurements

Secondary structure determination was carried out using a Jasco J-720 spectropolarimeter. Lyophilized peptide powders were dissolved in acetonitrile/PBS (1:2), and CD spectra were read immediately. Peptide concentrations were 20 μM for l-GLP-2 and 150 μM d-GLP-2 in acetonitrile/PBS (1:2). Samples were read using a 0.1 cm cuvette pathlength with three accumulations per run and 50 nm/min scanning speed. All spectra were background subtracted and converted to mean residue molar ellipticity using standard formulas to allow direct comparison between samples of varying concentration and amino acid length.

### Cell Lines and Reagents

HEK293 cell line was obtained from the American Type Culture Collection (ATCC; Rockville, MD). HEK293 cell line was tested for mycoplasma contamination. HEK293 cells were maintained in Dulbecco’s modified Eagle medium (DMEM) (ATCC) supplemented with 10% FBS and 1% pen/strep/glutamine, and the appropriate selection antibiotics, when required.

### HTRF cAMP Assay

HEK293 cells stably expressing hGLP-2R were trypsinized from subconfluent culture and seeded in a 96-well low volume plate (PerkinElmer, 66PL96025) at a density of 5000 cells per well. The cells were treated with different concentrations of l-GLP-2 peptide or d-GLP-2 peptides. After 1 h of incubation at 37 °C, cAMP d2 reagent and cAMP Eu-cryptate antibodies from the HTRF cAMP Gs Dynamic kit (PerkinElmer, 62AM4PEB) were added to each well. The plate was sealed and incubated for 30 min at room temperature. The samples were read using a luminometer with a 480 nm filter.

### β-Arrestin GPCR Assay

The PathHunter eXpress GLP-2R CHO-K1 β-arrestin-2 GPCR assay kit was purchased from Eurofins DiscoverX. Following the manufacturer’s instruction, the reporter cells were seeded in a total volume of 100 μL Assay Complete Cell Plating Reagent into white-walled, 96-well microplates. Different concentrations of l-GLP-2 peptide or d-GLP-2 analogues were treated in each well for 1 h at 37 °C. The assay signal was generated by adding the substrate, followed by a 1 h of incubation at room temperature. The samples were read using a BioTek Synergy 2 plate reader. The EC_50_ value of each peptide was calculated by fitting it to a non-linear sigmoidal curve using GraphPad Prism 8.

### Western Blot

HEK293 cells stably expressing hGLP-2R were treated with different concentrations (100 nM, 1 μM, and10 μM) of l-GLP-2 or d-GLP-2 peptides for specific time periods for p-AKT (0.5, 1, 3, and 6 h) and GFP expression (12, 24, 36, and 48 h). The cells were lysed with a lysis buffer 50 mM Tris-HCl pH 7.4, 1% Nonidet P-40, 150 mM NaCl, 1 mM ethylenediaminetetraacetic acid, 10 mM Na_3_VO_4_, 10 mM sodium pyrophosphate, and 25 mM NaF, 1× protease inhibitor mixture (Sigma) for 30 min at 4 °C. Protein samples were separated on a NuPage Bis·Tris 10% sodium dodecyl sulphate-polyacrylamide gel electrophoresis (SDS-PAGE) gel (Invitrogen) and transferred to polyvinylidene fluoride membranes. The transferred samples were immunoblotted with primary antibodies, followed by incubation with horseradish peroxidase-conjugated secondary antibodies (Santa Cruz Biotechnology) and detected using enhanced chemiluminescence (GE Healthcare).

### Protease Stability Assays

For proteinase K (ProtK; Bioshop) assay, stocks of 20 μM peptide in 200 μL of total volume (10 mM Tris-base, 10 mM NaCl, pH 7.4) were supplemented with 5 μM CaCl_2_, and 30 μL was removed for the un-treated T0 sample. Proteinase K (ProtK; Bioshop) was then added to a final concentration of 100 μg/mL. The samples were incubated at 37 °C, and 30 μL was removed after each time point, and protease activity was blocked by the addition of 10 mM phenylmethylsulfonyl fluoride (PMSF) (200 mM stock dissolved in isopropanol). Protease-inactivated samples were frozen at −20 °C until further use. Digestions were repeated three times. Frozen samples were supplemented with 8 μL of sample loading buffer (4× NuPAGE; Thermo Fisher Scientific), boiled (50 °C) for 10 min, and centrifuged (16,128*g*, 10 min) before loading the gel [12% NuPAGE Bis–Tris (Thermo Fisher Scientific)] with MES running buffer. Gels were run at 200 V for ∼35 min and stained using Coomassie brilliant blue dye. The densitometry of bands was determined using ImageJ software 5 with background subtraction. All samples were normalized to their respective untreated sample (T0).

For DPP-IV (ACROBiosystems) cleavage assay, 20 μM of peptides were diluted in 200 μL of total volume (100 mM Tris, pH 8.0), and 30 μL was removed for the untreated T0 sample. DPP-IV was then added to a final concentration of 50 μg/mL. Further samples were prepared following the above procedure to be analyzed by SDS-PAGE and mass spectrometry (Agilent 6538 UHD).

### Statistical Analysis

Statistical significance was analyzed by a two-tailed unpaired Student’s *t*-test using MS Excel. A *P* value of less than 0.05 was considered statistically significant.

## Supporting Information Available

Different sets of atom levels defined for each hotspot, quantification of the GFP and p-AKT levels induced for the d-GLP-2 analogues relative to l-GLP-2, sequences of the design retro-inverted d-GLP-2 analogues, d-GLP-2 downstream signaling, DPP-IV degradation experiments, and analytical characterization of the d-GLP-2 analogues (HPLC and mass spectrometry reports) (PDF)
Molecular formula strings (CSV)
PDB of d-GLP-2 analogues bound to GLP-2R (ZIP)

## Abbreviations

AKT: protein kinase B
cAMP: cyclic adenosine monophosphate
CAS: computational alanine scanning
CD: circular dichroism
CGI: Crooks Gaussian intersection
DMEM: Dulbecco’s modified Eagle medium
DMSO: dimethyl sulfoxide
d-GLP-2: glucagon-like peptide 2 d-peptide analogue
DPP-IV: dipeptidyl peptidase 4
EM: energy minimization
GLP-1: glucagon-like peptide-1
GLP-2: glucagon-like peptide-2
GLP-1R: glucagon-like peptide-1 receptor
GLP-2R: glucagon-like peptide-2 receptor
GPCR-Gs: G protein-coupled receptors
HPLC: high-pressure liquid chromatography
HTRF: homogeneous time-resolved fluorescence
EC_50_: half maximal effective concentration
l-GLP-2: glucagon-like peptide-2
MD: molecular dynamics
MS: mass spectra
NH2: amide group
NH–NH2: hydrazide group
PBS: phosphate-buffered saline
PDB: protein data bank
PKA: protein kinase A
PMSF: phenylmethylsulfonyl fluoride
PSM: palmitoylsphingomyelin
POPC: 1-palmitoyl-2-oleoylphosphatidylcholine, palmitoyloleoylphosphatidylcholine
ProtK: proteinase K
PTH: parathyroid hormone
RI: retro-inverse
RMSD: root mean square deviation
RMSF: root mean square fluctuation
SARS-CoV-2: severe acute respiratory syndrome coronavirus 2
