# Supplementary material for: Computational Design of Potent and Selective d-Peptide Agonists of the Glucagon-like Peptide-2 Receptor
Source: J Med Chem. 2023 Jul 25;66(15):10342–53. doi: 10.1021/acs.jmedchem.3c00464 (PMC10424673; doi:10.1021/acs.jmedchem.3c00464)
Supplement: Supplementary file 1 — jm3c00464_si_001.pdf [file jm3c00464_si_001.pdf]

Supporting Information

Computational Design of Potent and Selective D-peptide Agonists of the Glucacon-like Peptide 2 Receptor

Pedro A Valiente[1], Satra Nim[1], Jisun Kim[1], and Philip M Kim* [1, 2, 3]

[1] Donnelly Centre for Cellular and Biomolecular Research, University of Toronto, Toronto, ON M5S 3E1, Canada.
[2] Department of Molecular Genetics, University of Toronto, Toronto, ON M5S 3E1, Canada.
[3] Department of Computer Science, University of Toronto, Toronto, ON M5S 3E1, Canada.

[+] Both authors contribute equally

Corresponding author

*E-mail: pm.kim@utoronto.ca

Contents:

Table S1 Different sets of atom levels defined for each hotspot

Table S2 Quantification of the GFP and p-AKT levels induced for all the peptides relative to the L-GLP-2 treated.

Table S3 Sequences of the design retro-inverted D-GLP-2 analogs

Figure S1 D-GLP-2 peptides activate downstream signaling in time and dose-dependent manner. A-D) Western blots showing the levels of GFP expression and p-Akt, treated for the indicated time periods (A, C) and indicated dose concentrations (B, D). The relative band intensity of the p-Akt and GFP toward that of respective total protein was expressed as a percentage of that in the L-GLP-2, described in below the panel.

Figure S2 The D-GLP-2 analogs are highly resistant to DPP-IV cleavage. A) Sequence of GLP-2 and GLP-2G, a variant with the mutation A2G resistant to DPP-IV inactivation. The underlined residues at GLP-2 are cleaved by DPP-IV. Glycine at position 2 of GLP-2-2G is highlighted in red. **B**) Mass spectrum analysis of L-GLP-2 treated with DPP-IV after different incubation times. **C**) Mass spectrum analysis of D-GLP-2 treated with DPP-IV after different incubation times. **D)** Mass spectrum analysis of D-GLP-2 E33A treated with DPP-IV after different incubation times. **E)** Mass spectrum analysis of D-GLP-2 E33A hydrazide treated with DPP-IV after different incubation times. Each peptide's DPP-IV treatment incubation time is indicated above the mass spectrum analysis.

Figure S3 Analytical characterization of the D-GLP-2 peptide provided by the Lifetein company. A) HPLC report. As inset a table with the retention time of each peak **B)** Mass spectrometry report.

Figure S4 Analytical characterization of the D-GLP-2 E33A peptide provided by the Lifetein company. A) HPLC report. As inset a table with the retention time of each peak. **B)** Mass spectrometry report.

Figure S5 Analytical characterization of the D-GLP-2 E33A Hydrazide peptide provided by the Lifetein company. A) HPLC report. As inset a table with the retention time of each peak. **B)** Mass spectrometry report.

	Hotspots	Atom Levels	Atom types [a]
Helix1	H1	1	CB (J2), CG (J3), ND1 (JD1)
		2	CB (J2), CG (J3)
		3	CA (J1), CB (J2)
	F6	1	CB (W2), CG (W3), CD1 (WD1), CD2(WD1)
		2	CB (W2), CG (W3)
		3	CA (W1), CB (W2)
	E9	1	CD (Z3), OE1 (GE1)
		2	CG (Z2), CD (Z3)
		3	CB (Z1), CG (Z2)
		4	CA (Z0), CB (Z1)
Helix2	D8	1	CG (Z3), OD1 (CD1)
		2	CB (Z2), CG (Z3)
		3	CA (Z1), CB (Z2)
	L14	1	CG (Y3), CD1 (YD1) CD2 (YD1)
		2	CB (Y2), CG (Y3)
		3	CA (Y1), CB (Y2)
	L17	1	CG (Y3), CD1 (YD1) CD2 (YD1)
		2	CB (Y2), CG (Y3)
		3	CA (Y1), CB (Y2)
Helix3	D21	1	CG (Z3), OD1 (CD1)
		2	CB (Z2), CG (Z3)
		3	CA (Z1), CB (Z2)
	F22	1	CB (W2), CG (W3), CD1 (WD1), CD2(WD1)
		2	CB (W2), CG (W3)
		3	CA (W1), CB (W2)
	W25	1	CB (S2), CG (S3), CD2 (SD2)
		2	CB (S2), CG (S3)
		3	CA (S1), CB(S2)

Table S1 Different sets of atom levels defined for each hotspot

[a] Between parenthesis are the unique atoms name define for each atom in each hotspot residue

Table S2 Quantification of the GFP and p-AKT levels induced for all the peptides relative to the L-GLP-2 treated.

Peptides	p-AKT [a]	GFP [b]
D-GLP-2	1.58 ± 0.37	0.97 ± 0.16
D-GLP-2 E33A	1.74 ± 0.21	1.14 ± 0.15
D-GLP-2 E33A Hydrazide	2.28 ± 0.68	1.08 ± 0.14

a) p-AKT relative expression levels (mean ± sd, n =3) after 3 hours of incubation

b) GFP relative expression levels (mean ± sd, n =3) after 36 hours of incubation

Table S3 Sequences of the design retro-inverted D-GLP-2 analogs

Peptides	Full sequence[a]	Purity (%)	MW (g/mol)
**L-GLP2**	**H**GDGS**F**S**DE**MNTI**L**DN**L**AAR**DF**IN**W**LIQTKITD	95.19	3752.43
**D-GLP-2**	Acetyl-IKAQ**W**LAA**EF**IQAVAAV**L**AM**L**RNNQF**EF**AGVE**H**-NH2	95.10	3728.3
**D-GLP-2 E33A**	Acetyl-IKAQ**W**LAA**EF**IQAVAAV**L**AM**L**RNNQF**EF**AGVA**H**-NH2	96.60	3670.27
**D-GLP-2 E33A hydrazide**	Acetyl-IKAQ**W**LAA**EF**IQAVAAV**L**AM**L**RNNQF**EF**AGVA**H**-NH-NH2	95.04	3685

[a]The full sequence of each peptide is obtained by combining helix3+helix2+helix1. In bold are highlighted the residues that matched the selected hotspots in L-GLP2

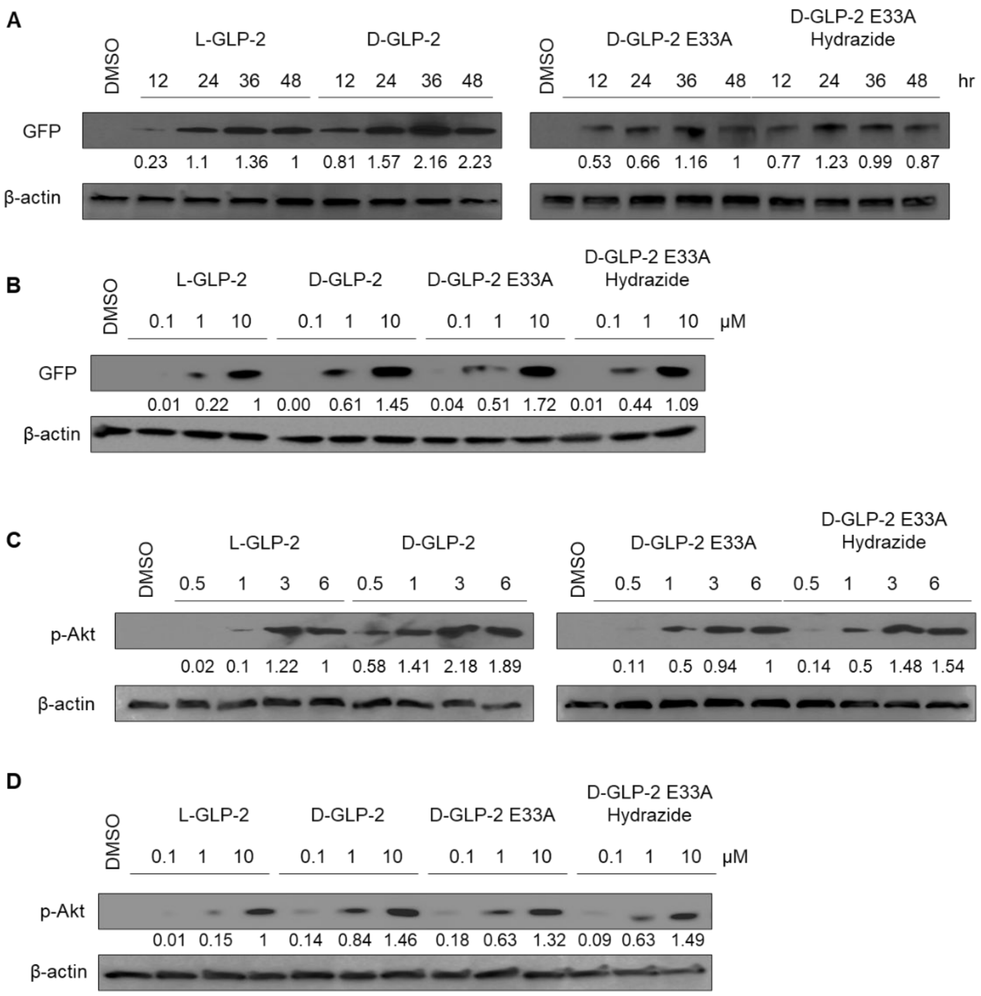

Figure S1 D-GLP-2 peptides activate downstream signaling in time and dose-dependent manner. A-D) Western blots showing the levels of GFP expression and p-Akt, treated for the indicated time periods (A, C) and indicated dose concentrations (B, D). The relative band intensity of the p-Akt and GFP toward that of respective total protein was expressed as a percentage of that in the L-GLP-2, described in below the panel.

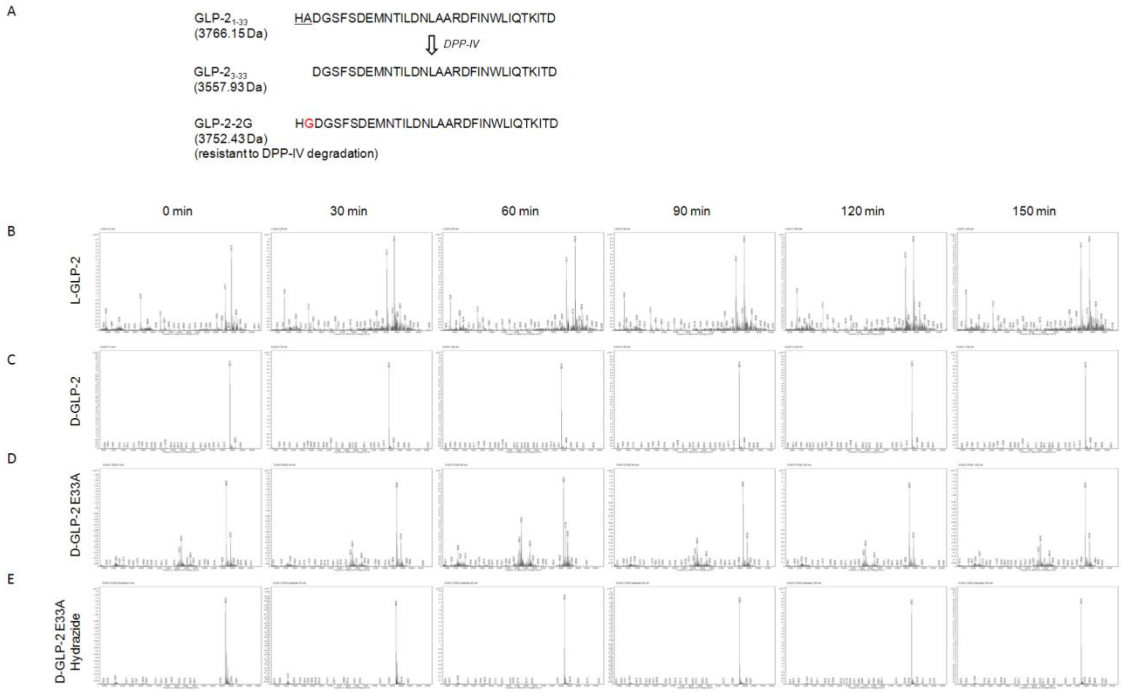

Figure S2 The D-GLP-2 analogs are highly resistant to DPP-IV cleavage. A) Sequence of GLP-2 and GLP-2G, a variant with the mutation A2G resistant to DPP-IV inactivation. The underlined residues at GLP-2 are cleaved by DPP-IV. Glycine at position 2 of GLP-2-2G is highlighted in red. **B**) Mass spectrum analysis of L-GLP-2 treated with DPP-IV after different incubation times. **C**) Mass spectrum analysis of D-GLP-2 treated with DPP-IV after different incubation times. **D)** Mass spectrum analysis of D-GLP-2 E33A treated with DPP-IV after different incubation times. **E)** Mass spectrum analysis of D-GLP-2 E33A hydrazide treated with DPP-IV after different incubation times. Each peptide's DPP-IV treatment incubation time is indicated above the mass spectrum analysis.

A

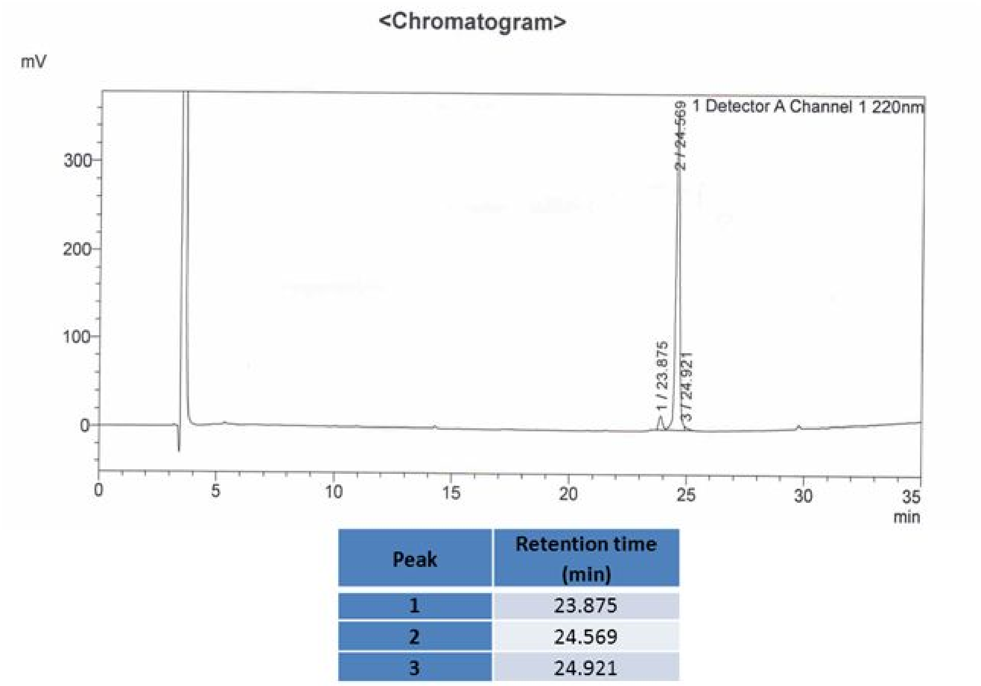

Peak	Retention time (min)
1	23.875
2	24.569
3	24.921

B

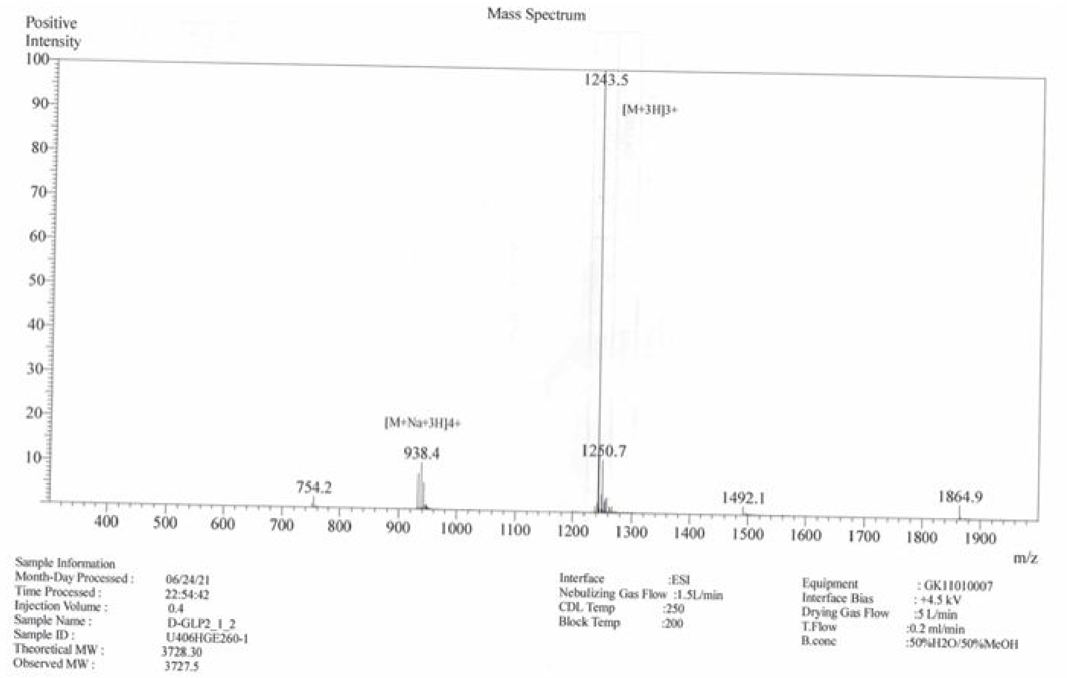

Figure S3 Analytical characterization of the D-GLP-2 peptide provided by Lifetein company. A) HPLC report. As inset a table with the retention time of each peak**. B)** Mass spectrometry report.

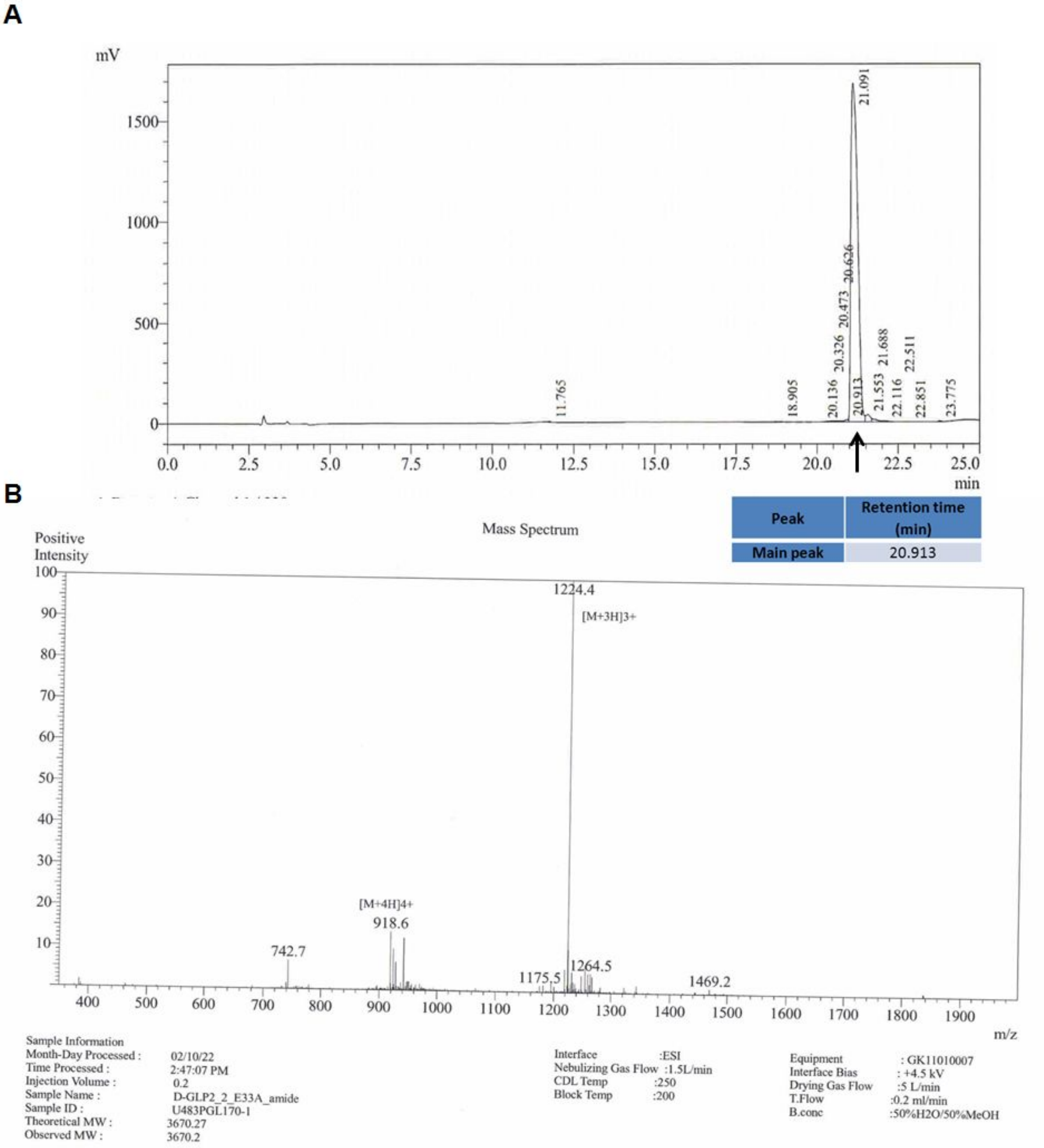

Figure S4 Analytical characterization of the D-GLP-2 E33A peptide provided by Lifetein company. A) HPLC report. As inset a table with the retention time of each peak**. B)** Mass spectrometry report.

A

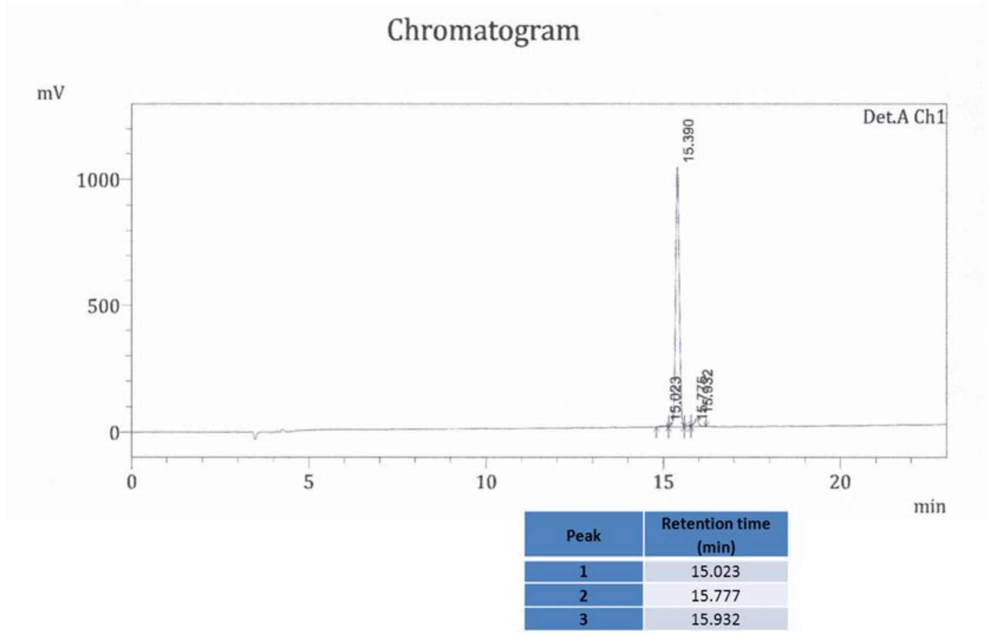

Peak	Retention time (min)
1	15.023
2	15.777
3	15.932

B

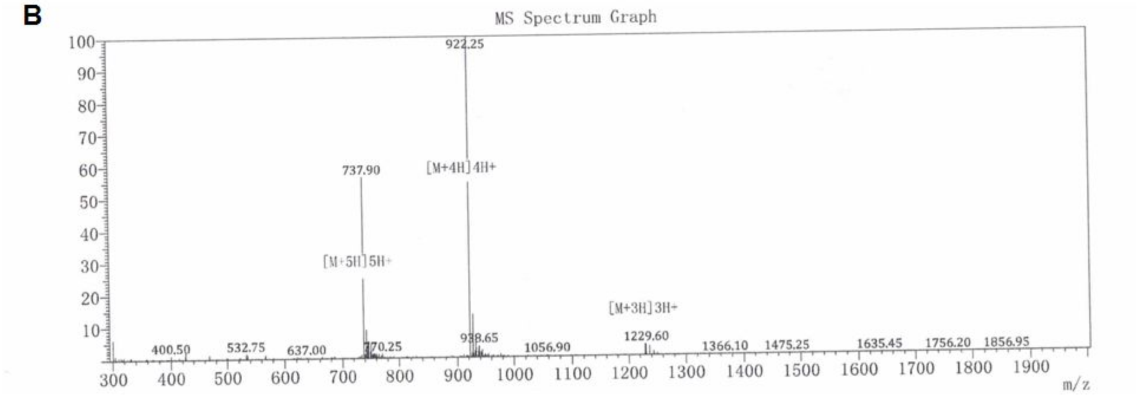

Figure S5 Analytical characterization of the D-GLP-2 E33A hydrazide peptide provided by Lifetein company. A) HPLC report. As inset a table with the retention time of each peak **B)** Mass spectrometry report.
